# Codeveloping an Online Resource for People Bereaved by Suicide: Mixed Methods User-Centered Study

**DOI:** 10.2196/56945

**Published:** 2025-01-20

**Authors:** Edouard Leaune, Kushtrim Bislimi, Pauline Lau-Taï, Héloïse Rouzé, Benoit Chalancon, Laurène Lestienne, Pierre Grandgenevre, Margot Morgiève, Nathalie Laplace, Guillaume Vaiva, Julie Haesebaert, Emmanuel Poulet

**Affiliations:** 1 Le Vinatier - Lyon Metropole Academic Hospital Center Bron France; 2 Research on Healthcare Performance (RESHAPE) National Institute for Health Research (INSERM U1290) University Claude Bernard Lyon 1 Lyon France; 3 Civil Hospices of Lyon Lyon Health Academic Center Lyon France; 4 CHRU Lille Centre Hospitalier Universitaire de Lille Lille France; 5 Medicine, Science, Health, Mental Health & Society Research Center (CERMES3) National Center for Scientific Research (CNRS UMR 8211), National Institute for Health Research (INSERM U988) Paris-Cité University Paris France; 6 Digital Agency Interlude Health Brignais France; 7 Lille Neurosciences & Cognition (LilNCog) National Institute for Health Research (INSERM U1172) Lille University Lille France; 8 Psychiatric Disorders, Neuroscience Research & Clinical Research (PSYR2) U1028, UMR5292 Lyon Neurosciences Research Center Bron France

**Keywords:** suicide bereavement, social media, mixed methods, participatory, user-centered, mobile phone, online resource, suicide, risk, suicidal behaviors, mental health, impairments, adaptive online resource, Information System Research, France

## Abstract

**Background:**

Although suicide bereavement is highly distressing and is associated with an increased risk of suicidal behaviors and mental and physical health impairments, those bereaved by suicide encounter difficulties accessing support. Digital resources offer new forms of support for bereaved people. However, digital resources dedicated to those bereaved by suicide are still limited.

**Objective:**

This paper aimed to develop and implement an evidence-based, innovative, and adaptive online resource for people bereaved by suicide, based on their needs and expectations.

**Methods:**

We performed a mixed methods, participatory, user-centered study seeking to build resources from the perspectives of people bereaved by suicide and professionals or volunteers working in the field of postvention. We used the Information System Research framework, which uses a three-stage research cycle, including (1) the relevance cycle, (2) the design cycle, and (3) the rigor cycle, and the Design Science Research framework.

**Results:**

A total of 478 people participated in the study, including 451 people bereaved by suicide, 8 members of charities, and 19 mental health professionals working in the field of postvention. The development stage of the resource lasted 18 months, from October 2021 to March 2023. A total of 9 focus groups, 1 online survey, 30 usability tests, and 30 semistructured interviews were performed. A website for people bereaved by suicide named “espoir-suicide” was developed that includes (1) evidence-based information on suicide prevention and bereavement, (2) testimonies of people bereaved by suicide, (3) a delayed chat to ask questions on suicide and bereavement to a specialized team of mental health professionals, and (4) an interactive nationwide resource directory. The mean system usability score was 90.3 out of 100 for 30 participants, with 93% (n=28) of them having a rating above 80. Since the implementation of espoir-suicide in March 2023, a total of 19,400 connections have been recorded, 117 local resources have been registered nationwide, and 73 questions have been posted in the chat.

**Conclusions:**

The use of a mixed methods, participatory, user-centered design allowed us to implement an evidence-based, innovative, and functional website for people bereaved by suicide that was highly relevant for fulfilling the needs and expectations of French people bereaved by suicide.

**International Registered Report Identifier (IRRID):**

RR2-10.3389/fpsyt.2021.770154

## Introduction

Each year, approximately 700,000 individuals die by suicide worldwide [1]. Approximately 7 million relatives are bereaved by suicide in the aftermath [2]. Suicide bereavement is highly distressing and is associated with an increased risk for suicidal behaviors, as well as mental and physical health impairments [3,4]. Nevertheless, those affected by suicide bereavement encounter unmet needs, including difficulties accessing informal or formal support after their bereavement [4,5]. The social stigma surrounding suicidal behaviors and bereavement is associated with high levels of loneliness and lack of social support [6]. In France, the suicide rate has been on a downward trajectory for more than 30 years, although it remains higher than the global and European averages [7]. In 2014, the government introduced a national suicide prevention strategy, which has yielded encouraging outcomes. However, the resources available to those who have lost a loved one to suicide remain limited, prompting the exploration of digital resources as a potential avenue for supporting suicide survivors in France.

Digital resources are currently changing the way we experience death and grief by offering new forms of support to people who have lost loved ones [8]. They include online forums, online support groups, mailing groups, online memorials, or online interventions such as online peer-support groups or online psychotherapeutic counseling [9]. A recent systematic review on 11 studies demonstrated that the internet and social networks are frequently used by individuals bereaved by suicide [9], as online resources promote 24×7 access to help and support for everyone, including people who can be far from health care services. Furthermore, it offers some benefits, such as easy and unlimited access, anonymity, a nonjudgmental space, and direct access to other people bereaved by suicide [9]. For example, Kramer et al [10] reported in a longitudinal cohort of 270 participants in the Netherlands a significant increase in well-being and a decrease in depressive symptoms at 12 months after using an online forum for people bereaved by suicide. In Sweden, Westerlund [11] conducted a cross-sectional survey in Sweden on 370 participants to investigate potential predictors of satisfaction with psychosocial health after using an online support group or an online memorial for suicide bereavement. Online support group activity was found to be significantly associated with satisfaction regarding psychosocial health, while memorial website activity showed a tendency to a negative association. A recent German study also reported that completing an online group intervention for the suicide bereaved could significantly reduce trauma-related outcomes [12].

However, resources dedicated to those bereaved by suicide are still limited, and the assessment of their relevance and effectiveness remains scarce [9,13]. In addition, to our knowledge, none of the existing resources have been designed or implemented based on an evidence-based process involving participants with lived experience of suicide bereavement [9]. However, the participation of people with lived experience in research, especially concerning mental health and suicide bereavement, is essential for developing a well-accepted and effective postvention tool reaching the needs and expectations of people bereaved by suicide [14].

Hence, the main objective of our study was to develop and implement an innovative and ergonomic online resource for French people bereaved by suicide, aligned with their needs and expectations toward digital resources after bereavement.

## Methods

### Overview

The protocol of the study has been exhaustively described in a study by Leaune et al [15]. We conducted a mixed methods, participatory, user-centered study seeking to build resources from the perspectives and needs of people bereaved by suicide and professionals or volunteers working in the field of postvention. We used the Information System Research (ISR) framework to guide the design of the study [16]. The ISR framework uses various design processes to build a product or design an artifact, such as a mental health online resource. According to the literature on the ISR [16], a three-stage research cycle was planned, including (1) the relevance cycle, (2) the design cycle, and (3) the rigor cycle. Our 3-stage research cycle also aligns with the Design Science Research (DSR) framework, which is defined as “a qualitative research approach in which the object of study is the design process, i.e. it simultaneously generates knowledge about the method used to design an artifact and the design or the artifact itself” [17]. The DSR has been demonstrated to be an effective approach for the development of mobile health digital resources. It involves a series of cyclical steps, beginning with the identification of a problem and ending with the communication of the solution [18]. These steps include defining the solution, establishing the solution objective, developing and evaluating the solution, and demonstrating it. Our study aligns perfectly with the DSR methodology. We used a combination of qualitative and quantitative data to identify the needs of people bereaved by suicide; define the solution and its objective; and develop, evaluate, and communicate an adapted solution. The study also aimed to generate insights into the methodology used, the efficacy of a participatory approach, and the challenges encountered in designing an online resource in the context of bereavement and mental health.

### Mixed Methods Evaluation

Mixed methods studies have been shown to be effective in evaluating the implementation of new prevention and intervention programs in mental health [19]. A mixed methods design focuses on collecting, analyzing, and merging both quantitative and qualitative data into one study. According to the taxonomy of mixed methods studies described by Palinkas et al [19], the structure of the study relied on a sequential and complementary collection of qualitative and quantitative data.

### User-Centeredness

The participation of people bereaved by suicide in the 3 cycles of the study aimed to ensure the user-centeredness of both the resource development and the research process. Their active participation throughout the entire research and design or implementation process was expected to offer a unique means to develop an innovative and adaptive resource that meets their needs and expectations. The participation of the other stakeholders (health professionals, associations, and peers) was expected to offer a better understanding of bereaved people’s needs. The coproduction of information on suicide bereavement and digital resources enables users to use and distribute their experiential knowledge, skills, and expertise with researchers and other participants [20]. By interacting, the team inaugurated a culture of empowering practices that let users freely express themselves and make impactful contributions to the research process [21]. The study’s participatory design was ensured through the establishment of an environment promoting equality, equity, and empowerment.

### Inclusion and Exclusion Criteria

French adults bereaved by suicide were eligible to participate in the 3 stages of the study, whatever the duration since loss and the kinship with the deceased. Health care professionals, social workers, or volunteers working in postvention units or charities dedicated to bereavement were also eligible for participation in stages 1 (excluding the survey) and 2 (focus groups). They should have been at work or volunteering in the field of suicide bereavement and postvention for at least 1 year. People younger than 18 years of age and people bereaved by causes other than suicide were excluded from the study.

The survey (stage 1) and all the focus groups (stages 1, 2, and 3) were administered online to include people nationwide. In stage 3, some of the user tests were conducted on-site, and the other test was conducted under ecological conditions on their own devices from their homes. The tests were conducted either on smartphones or on a computer.

For the 3 stages, participants were recruited through social media and charities dedicated to suicide bereavement. A purposive snowballing recruitment process was used to construct a convenience sample covering a broad range of sociodemographic profiles. No incentive was offered for participation in the study.

### Data Collection

**Figure 1:**
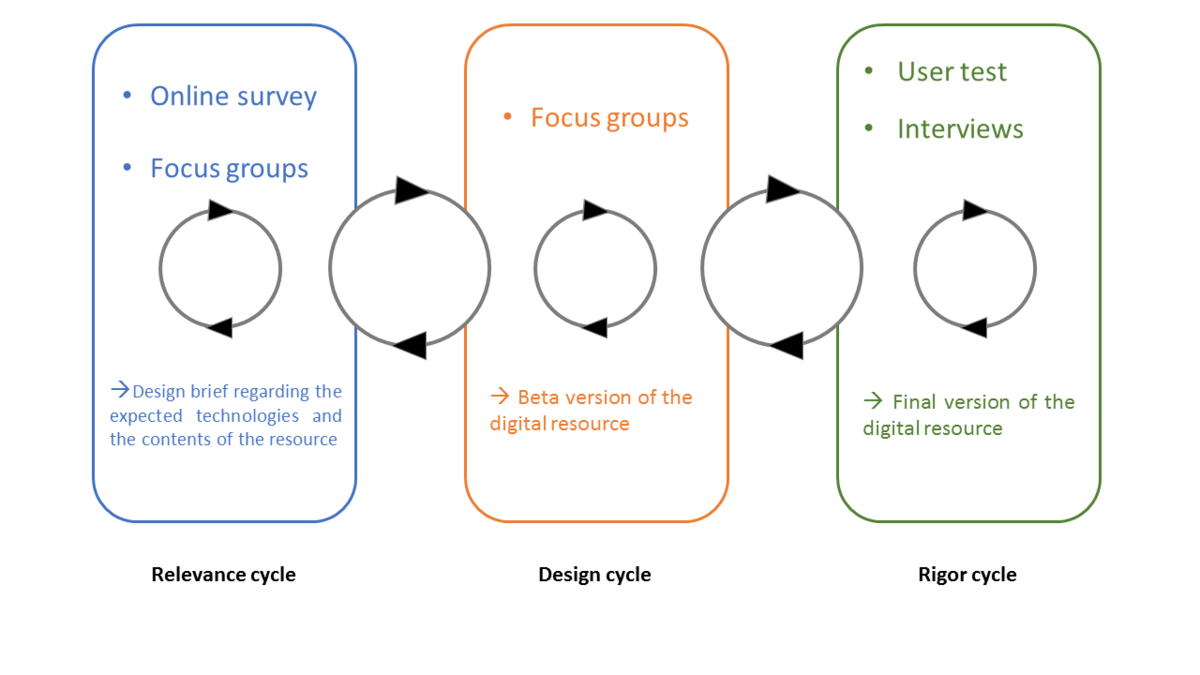
The Information System Research framework used in the ESPOIR2S study.

According to the mixed methods research design of the study, quantitative and qualitative data were sequentially and complementarily collected through a 3-stage process to obtain an in-depth evaluation of the implementation of the online resource. Quantitative and qualitative data were collected and analyzed during (1) the relevance cycle through an online questionnaire and focus groups, (2) the design cycle through focus groups, and (3) the rigor cycle through an online questionnaire and semistructured interviews (Figure 1).

#### Stage 1: Relevance Cycle

The relevance cycle aimed to understand the environment of the end user by determining requirements. The needs and expectations of people bereaved by suicide, professionals, and volunteers working in the field of postvention were collected through an online questionnaire and a series of focus groups.

A 26-item online questionnaire was built according to a recent systematic review of digital resources for suicide bereavement [9] and was hosted on the website Lime Survey. The questionnaire collected sociodemographic characteristics and evaluated four dimensions: (1) perceived needs regarding suicide bereavement, (2) use of online resources associated after suicide loss, (3) needs and expectations regarding the development of online digital resources for people bereaved by suicide, and (4) personal propositions regarding the development of online digital resource for people bereaved by suicide. We determined that the participation of 385 respondents would permit us to estimate a proportion with a 95% CI and a 5% error margin, as determined in a previous study [22]. The questionnaire was subsequently sent to participants through social media and through associations for people bereaved by suicide. Purposive snowballing sampling was used to access the link to the online questionnaire to the maximum number of putative participants.

The focus groups included 3 categories of participants—people bereaved by suicide, postvention professionals, and volunteers working in the field of bereavement and postvention. To obtain the perspectives of all the stakeholder profiles, we adopted a maximum variation recruitment process to constitute our focus groups. To facilitate the participatory process, each focus group was led by 2 researchers (a facilitator and an assistant; KB and PL-T) and organized through the process, such as (1) work objectives, (2) emerging needs and expectations, (3) hypotheses and concepts, (4) putative solutions, and (5) conclusions [23]. No guide was used for the focus groups. The content of the focus group was recorded and anonymized. The needs and expectations of people bereaved by suicide were collected and synthesized to construct a brief overview of digital resources. The data collected during the relevance cycle aimed to inform the design brief regarding the expected technologies and the contents of the resource.

#### Stage 2: Design Cycle

The design cycle aims to produce digital resources by compiling qualitative and quantitative data collected during the relevance cycle through an iterative process of building and evaluating digital resources.

Focus groups were established with people bereaved by suicide, members of associations for bereaved people, postvention professionals, and web designers from a startup specialized in eHealth solutions. Again, each focus group was led by 2 researchers (KB and PL-T) with no guidance, which led to the redaction of specifications or adjustments of the resource, based upon which the web designers progressively and iteratively designed a prototype of the digital resource. The iterative feedback of participants on the resource and its final validation ensured the incremental design of an online resource adapted to the needs and expectations of people bereaved by suicide and stakeholders. The content of the focus group was recorded and anonymized. A pilot of the online resource was expected to be designed and built at the end of the design cycle before its evaluation during the rigor cycle.

#### Stage 3: Rigor Cycle

The rigor cycle served as the final evaluation and aimed to evaluate the acceptability and the perceived effectiveness of the online resource by the users.

##### Quantitative analysis

The French version of the System Usability Scale (SUS) [24] was used to assess (1) satisfaction with the resource and (2) acceptability of its design and content. The SUS is a 10-item Likert-scale questionnaire that provides a global view of the subjective assessment of a system’s usability [24]. The SUS scores range from 0 to 100 (scores range from 0 to 10 for each item) [24]. A score above 70 indicates good acceptability of a resource, and a score above 80 indicates very good acceptability [25]. We concluded that acceptability was reached if at least 80% (24/30) of the respondents rated the acceptability of the SUS above 80.

##### Qualitative analysis

Semistructured interviews were also performed with the participants to qualitatively assess the accessibility and perceived effectiveness of the resources. The participants were questioned about their perception of the online resource (satisfaction, usability, and acceptability) and the putative modifications that they may recommend.

We also used a heuristic evaluation, which offers a quick and methodical way to identify and categorize usability problems. The participants were asked to follow scenarios when using the resource (ie, going back to the main page, finding specific content, and using specific options). To create these scenarios and to analyze the user tests, the Scapin and Bastien [26] ergonomic criteria for human-computer interfaces were used. These criteria, namely guidance, workload, explicit control, adaptability, error management, consistency, significance of codes, and compatibility, were qualitatively assessed during the heuristic evaluation and used to improve the platform.

##### Sample

According to the previous literature on the SUS [27,28], a total of 30 people bereaved by suicide were expected to participate in the rigor cycle. The combination of quantitative and qualitative analyses of acceptability enabled us to identify the strengths and weaknesses of our online resources to proceed (or not) further with improvements and with a larger-scale effectiveness evaluation study.

The final evaluation of acceptability and usability was based on the combination of SUS results and heuristic evaluation. The final version of the online resource was designed and built at the end of the rigor cycle before its implementation.

### Additional Analyses

In order to assess the implementation and use of the online resource, a series of key performance indicators were monitored 18 months after its launch. These included the number of visits to the online resource, the number of local resource records on the platform, and the number of questions posted on the chat.

### Data Analysis

The qualitative results were combined with the quantitative results to provide a detailed overview of the acceptability of the online resources.

#### Quantitative Analysis

Data manipulation and analyses were performed using R software (version 3.4.1; R Core Team). Categorical variables are summarized using numbers and percentages, and quantitative variables are described using either means and SDs or medians and IQRs. For stage 1, the number of respondents, number of completed questionnaires, characteristics of respondents (age, gender, socioeconomic status, and relation to the deceased), and responses to the questionnaire were analyzed using descriptive statistics. Subgroup analyses of responses according to age category (defined according to the distribution of age of the respondents), sex, and relationship to the deceased were also conducted. A report summarizing the results was produced and presented during the focus groups. For stage 3, the characteristics of the included population were described. The main outcome criterion was the SUS score. The SUS score was described as a continuous variable by its mean (SD) and median (IQR). SUS was dichotomized as SUS>80 (very good usability) versus SUS<80 (moderate or poor usability) [25].

#### Qualitative Analysis

Based on the verbatims of the focus groups, semistructured interviews, or heuristic evaluation, a content analysis was performed, with several chronological phases (reanalysis, operation of equipment, and interpretation) [29]. Furthermore, 2 authors (KB and PL-T) first looked at the apparent messages through a repeated reading of the transcripts to achieve immersion and obtain a sense of the whole. In addition, this first reading allowed us to define the thematic and formal categories relevant for later coding speeches. Units of meaning were then independently identified, categorized, and put into relation to identify axes of transversal meanings. This process allowed us to classify the elements and construct a simplified representation of the raw data to obtain further quantitative data to better understand their needs.

#### Merging Quantitative and Qualitative Data

For the relevance cycle (stage 1), the use of qualitative and quantitative analyses provided a comprehensive understanding of the development and evolution of the needs and expectations of people bereaved by suicide and stakeholders. For the rigor cycle (stage 3), the addition of user tests using SUS and semidirected interviews permitted us to observe the problems, if any, encountered on the platform in different concrete situations.

### Ethical Considerations

The study received ethical approval from the Ethical Review Board of The University Claude Bernard Lyon 1 (institutional review board approval 2021-01-12-04). The data were anonymized, and compensation was provided for travel expenses in connection with participation in the study.

## Results

### Total of Participants

A total of 478 people participated in the study, including 451 (94.3%) people bereaved by suicide, 19 (3.9%) mental health professionals working in the field of postvention, and 8 (1.7%) members of charities. The development stage of the resource lasted 18 months, from October 2021 to March 2023.

### Relevance Cycle

A total of 417 people participated in the relevance cycle from October 2021 to February 2022; 401 participated in the online survey, and 16 participated in the focus groups.

#### Online Survey

A total of 401 people bereaved by suicide participated in the online survey. The results of the online survey have been exhaustively described elsewhere [30]. The characteristics of the participants are reported in Table 1. Most of them were women bereaved by the suicide of their child. A slight majority of the participants had been bereaved for less than 3 years and benefited from counseling during their bereavement process. The mean age was 45.7 years; the age range was 18 to 80 years.

**Table 1:** Characteristics of the participants in the survey of the relevance cycle.

Characteristics			Values (n=401), n (%)
**Age (years), mean (SD)**			45.7 (12.7)
**Gender**			
	Women	354 (88.3)	
	Men	46 (11.5)	
	Nonbinary	1 (0.2)	
**Status of the deceased**			
	Child	133 (33.2)	
	Sibling	56 (14)	
	Partner	59 (14.7)	
	Parent	56 (14)	
	Other	97 (24.2)	
**Duration of bereavement**			
	>3 years	201 (50.1)	
	<3 years	200 (49.9)	
**Counseling**			254 (63.3)
	Counseling by psychotherapist^a^	129 (32.2)	
	Counseling by a psychologist	99 (24.7)	
	Counseling by a psychiatrist	72 (18)	
	Group therapy	22 (5.5)	
	Counseling in a charity	71 (17.7)	
**Frequency of internet use**			
	Daily	369 (92.5)	
	Weekly	24 (6)	
	Occasionally	6 (1.5)	
**Frequency of social media use**			
	Daily	308 (77.6)	
	Weekly	48 (12.1)	
	Occasionally	41 (10.3)	
**Available digital devices**			
	Computer	298 (74.3)	
	Smartphone	379 (94.5)	
	Digital tablet	102 (25.4)	

^a^In France, psychotherapists are neither psychiatrists nor psychologists.

Nearly three-quarters (296/401, 73.8%) of the sample used the internet for their grief process, while nearly two-thirds (247/401, 61.6%) used social media. Regarding social media, Facebook (Meta) was used by the majority of participants (233/401, 58.1%). The participants mostly reported their use of the internet or social media as beneficial or very beneficial after bereavement—33.1% (98/296) for the internet and 31.6% (78/247) for social media. At the same time, few participants reported their use as not beneficial—9.8% (29/296) for the internet and 13.4% (33/247) for social media. The participants mainly used the internet to find information on suicide (286/296, 96.6%) and to access testimonies of other people bereaved by suicide (231/296, 78%). The use of social media was associated mainly with honoring the memories of their relative deceased by suicide (144/247, 58.3%%), accessing testimonies of other people bereaved by suicide (137/247, 55.5%), and discussing with other people bereaved by suicide (116/247, 47%).

A web platform (281/401, 70.1%) or a specific social media platform (232/401, 57.9%) was expected to be developed by the majority of the participants. Information on suicide bereavement (261/401, 65.1%) and suicide prevention (240/401, 59.9%) were the most reported expectations regarding digital resources. Discussion with a mental health professional was also frequently expected (229/401, 57.1%), while accessing online counseling was the least frequently reported expectation (109/401, 27.2%). Access to testimonies by other people bereaved by suicide was expected by a slight majority of the participants (214/401, 53.4%). When compiling expectations in 4 dimensions, obtaining information on suicide and suicide bereavement (310/401, 77.3%) and reaching peers bereaved by suicide (263/401, 65.6%) were the most frequently reported expectations.

#### Focus Groups

A total of 3 focus groups were performed, including 16 people, 4 of whom were bereaved by suicide, 8 of whom were professionals working in the field of postvention, and 4 of whom were members of charities. Each focus group lasted 1-2 hours and included 4-8 participants. Through the three focus groups, the participants agreed that an online digital resource for people bereaved by suicide should (1) offer validated and relevant information on suicide, suicide prevention, and bereavement; (2) provide guidance to people bereaved by suicide; (3) be personalized and anonymous; (4) centralize existing and validated knowledge on suicide, suicide prevention, and bereavement; and (5) centralize existing resources for people bereaved by suicide.

### Design Cycle

A total of 31 people participated in a total of 6 focus groups during the design cycle from February 2022 to September 2022; a total of 16 people were bereaved by suicide, 11 professionals worked in the field of postvention, and 4 professionals were members of organizations. Each focus group lasted 1-2 hours and included 4-8 participants. The focus groups mainly relied on topics, such as (1) design of the platform—feedback on the mockup of the platform (n=3) and design of the mockup (n=6); (2) content of the platform—naming the different functions (n=4), organizing information on the platform (n=3), and delayed chat (n=6). Some of these focus groups could have more than 1 topic because of the iteration process needed in a user-centered conception.

**Figure 2:**
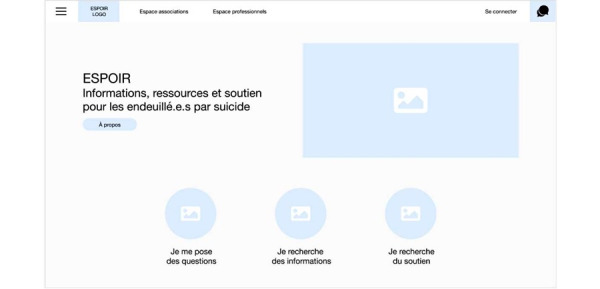
Screenshot of the beta version. English translations: “Informations, ressources et soutien pour les endeuillé.e.s par suicide”: Information, resources and support for people bereaved by suicide; “Á propos”: About; “Je me pose des questions”: I ask myself some questions; “Je recherche des informations”: I'm looking for information; “Je recherche du soutien”: I'm looking for support.

According to the results issued from the relevance cycle and from the 6 focus groups performed during the design cycle, a website was chosen for people bereaved by suicide, including features such as (1) evidence-based information on suicide prevention and bereavement, (2) testimonies of people bereaved by suicide, (3) a delayed chat to ask questions on suicide and bereavement to a specialized team of mental health professionals, and (4) an interactive nationwide resource directory. The authors selected the website “espoir-suicide” (“ESPOIR” means “hope” in French and is an acronym for People bereaved by Suicide: Platform for Orientation, Information and Resources [*Endeuillés par Suicide: Plateforme d’Orientation, Informations et Ressources*]) and designed it to offer easy access and navigation for people bereaved by suicide at any stage after the death of their relative. It also offers easy access to the national helpline for suicide prevention (3114). The beta version of the platform was built at the end of the design cycle (Figure 2) before it was evaluated by a panel of people bereaved by suicide.

### Rigor Cycle

A total of 30 people bereaved by suicide participated in the rigor cycle from September 2022 to March 2023. Participant characteristics are displayed in Table 2. Among them, 26 were women and 20 were bereaved by the suicide of their child. The mean age was 53.5 (SD 14.7) years. The mean SUS score after the user tests was 90.3, with 93% (28/30) of the participants rating the SUS above 80.

**Table 2:** Characteristics of the participants in the rigor cycle.

Participant	Gender	Age (years)	Status the deceased	Time since loss (years)	Test device	SUS^a^ score
P1	Woman	23	Sibling	2	Smartphone	92.5
P2	Woman	34	Sibling	1.5	Computer	92.5
P3	Woman	66	Child	16	Computer	92.5
P4	Man	56	Child	7	Computer	97.5
P5	Woman	56	Child	7	Computer	87.5
P6	Woman	21	Parent	2.5	Computer	92.5
P7	Woman	42	Partner	0.3	Computer	62.5
P8	Woman	74	Partner	26	Computer	72.5
P9	Woman	24	Best friend	5.5	Computer	95
P10	Man	75	Child	14	Computer	95
P11	Woman	61	Child	8	Computer	97.5
P12	Woman	67	Child	14	Computer	87.5
P13	Woman	41	Child	1.5	Smartphone	95
P14	Woman	48	Partner	1	Smartphone	82.5
P15	Woman	56	Child	1.5	Computer	85
P16	Woman	51	Child	0.2	Computer	95
P17	Woman	51	Partner	1	Computer	97.5
P18	Woman	68	Child	12	Computer	92.5
P19	Woman	60	Child	2.5	Computer	85
P20	Woman	46	Partner	3	Computer	90
P21	Woman	63	Child	0.5	Computer	87.5
P22	Man	59	Child	0.5	Computer	80
P23	Woman	57	Child	8	Computer	92.5
P24	Woman	36	Friend and grandparent	1 and 12	Computer	95
P25	Woman	66	Child	5	Computer	100
P26	Woman	66	Partner	1	Computer	90
P27	Woman	65	Partner	3	Computer	87.5
P28	Man	68	Child	12	Computer	97.5
P29	Woman	54	Child	3.5	Computer	97.5
P30	Woman	52	Children (lost 2 daughters)	5 and 6	Computer	96

^a^SUS: System Usability Scale (score ranging from 0 to 100).

Each interview and heuristic evaluation lasted between 30 and 60 minutes. The participants reported 3 main strengths of the espoir-suicide platform in terms of guidance, workload, consistency, adaptability, and explicit control:

1. The aesthetics of the website

I truly love the colors. I find it sweet, it’s calming. It’s truly important when you go on a site like this.

P11

The small side springs out on the 3 features; it gives a dynamic feel; there’s that bounce-back feel.

P16

2. The main functions

I cannot wait to read the answers to the chat. It lacked a site like this; it is great that professionals are answering.

P25

It is very, very good. I will not hide the fact that I will go back when it goes online. Looking at it like this, it seems very complete.

P19

3. Access to testimonies

It’s important to see that we are not alone, to see others live the same thing as me.

P10

It is good that there’s a little summary so that people can find what they are looking for.

P11

Some of the participants also reported the following limitations and needs for improvement, in terms of significance of codes, error management, and compatibility:

1. Access to home page

I find there’s a problem on all the pages; there’s no back button.

P7

2. Visibility of favorite options

I cannot see where to click for the favorite options.

P8

3. Presentation of the national helpline for suicide prevention (3114)

Maybe mention it too. I’m saying this because we need to be reassured about confidentiality and anonymity. If people need to know more, there should be a small link to the national suicide helpline.

P14

4. Need to simplify the names of some subtopics.

I did not understand “risk factors”. Maybe put more words. The same goes for “signs of distress“; it is not clear enough.

P24

The espoir-suicide website was improved according to their remarks.

### Implementation

**Figure 3:**
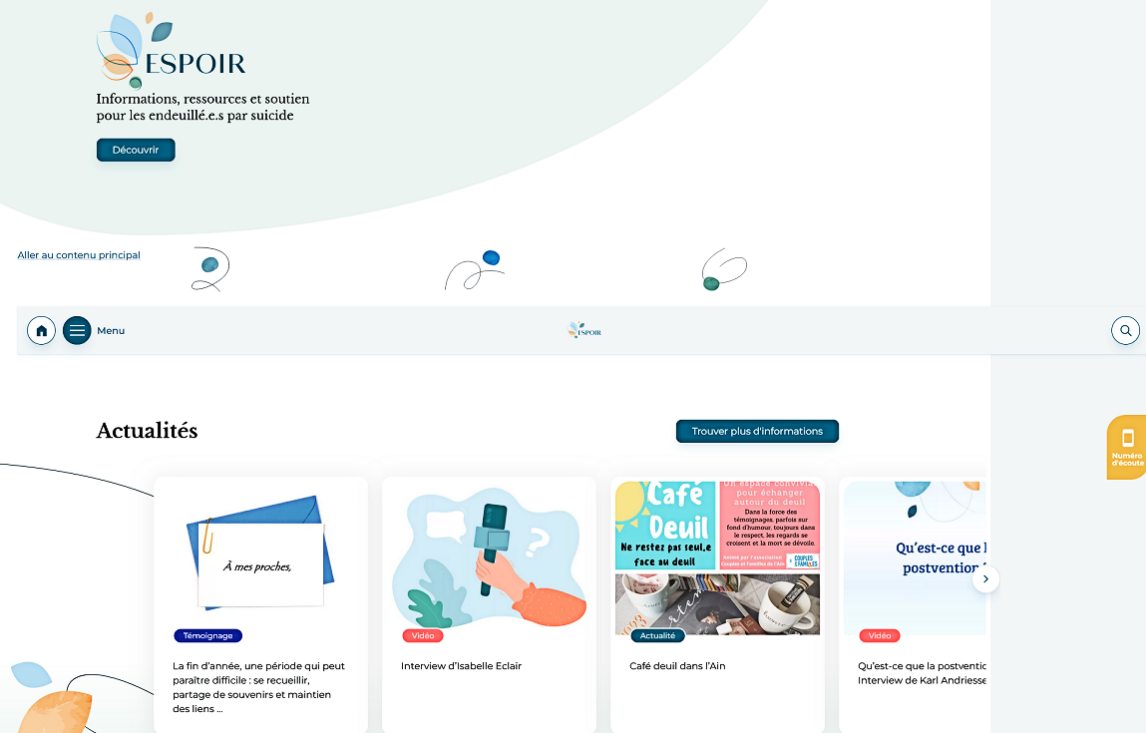
Screenshot of the main page of the espoir-suicide platform. English translations: “Informations, ressources et soutien pour less endeuillé.e.s par suicide”: Information, resources and support for people bereaved by suicide; “Découvrir”: Discover; “Aller au contenu principal”: Go to main content; “Actualités”: News; “Trouver plus d’informations”: Find more information; “Á mes proches”: To my loved ones; “Témoignage”; Testimony; “La fin d’année, une période qui peut paraître diffcile : se recueillir, partage de souvenirs et maintien des liens”: “The end of the year, a period that can seem difficult: reflecting, sharing memories and maintaining connections”; “Interview d'Isabelle Eclair”: Interview with Isabelle Eclair; “Café deuil dans l'Ain”; Mourning café in Ain.

**Figure 4:**
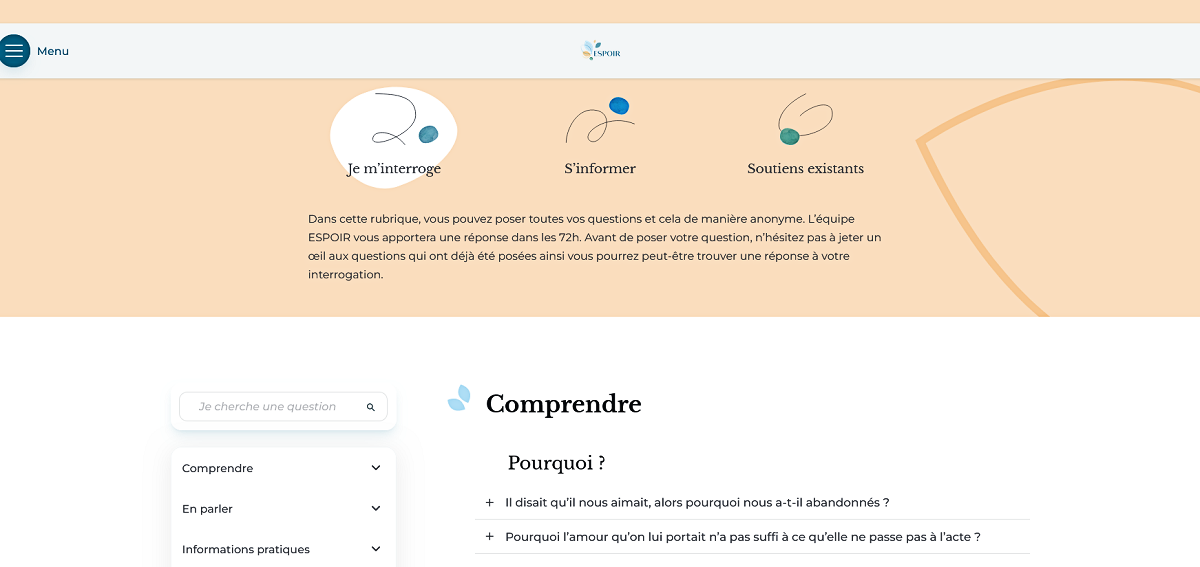
Screenshot of the chat. English translations: “Je m’interroge”: I'm wondering; “S’informer”: Get informed; “Soutiens existants”: Existing support; “Dans cette rubrique, vous pouvez poser toutes vos questions et cela de manière anonyme. L'équipe ESPOIR vous apportera une réponse dans les 72h. Avant de poser votre question, nhésitez pas à jeter un oeil aux questions qui ont déjà été posées, ainsi vous pourrez peut-être trouver une réponse à votre interrogation”: In this section, you can ask all your questions anonymously. The ESPOIR team will get back to you within 72 hours. Before sending your question, don't hesitate to have a look at the questions that have already been posted, so you may find an answer to your question; “Comprendre”: Understand; “En parler”: Talk about it; “Informations pratiques”: Practical information; “Pourquoi ?”: Why?; “Il disait qu’il nous aimait, alors pourquoi nous a-t-il abandonnés ?”: He said he loved us, so why did he leave us?; “Pourquoi l'amour qu'il nous portait n'a pas suffi à ce qu'elle ne passe pas à l'acte”: Why the love she had for us was not enough to keep her from acting on it.

The online platform “espoir-suicide” was launched in March (Figures 3 and 4). The platform was launched through media, including national newspapers, and through mental health services and charities.

Since the implementation of the resource, a total of 20,600 visits were recorded. A total of 122 local resources (including health care settings, charities, counselors, and so on) all around the country were registered on the platform to provide help and support to people bereaved by suicide in their department or region. A total of 81 questions were posed, mainly in the evenings, to the mental health team by people who were recently or long-term bereaved, covering a wide range of suicide bereavement issues (emotional effects, disclosure of suicide bereavement with others, discussions about suicide within the family, and so on). Answers were given by the mental health team 72 hours after they were posted. Here are 3 examples of questions posted in the chat:

My daughter was also a sister, a companion, a granddaughter, a cousin, a godmother and a friend. Does the mother-child bond and relationship result in a more specific and more tragic form of grief than that experienced in other contexts?

My brother died by suicide 13 years ago. There’s a photo of him and me at home when we were little and my daughter, who is 9, has now realized that it was my brother in the photo. She’s started asking questions, and I have answered honestly, explaining that he died by suicide, but she’s asking very specific questions about why he did it, how he did it, who found him (I was the one who found him). I do not know how I’m supposed to answer these questions, how much detail I can give, because it seems violent for a child her age. Thank you for your help.

When my friend died by suicide a year ago, I told the people around me that he had died in a road accident because there had already been suicides in my family and I did not want to make things worse. Is there any point in telling the truth? Thank you for your reply.

## Discussion

### Summary of Results

We performed the first mixed methods, participatory, user-centered study aimed at developing an online digital resource for people bereaved by suicide. Our findings support the efficiency of codevelopment research through the ISR framework for developing digital resources for mental health, especially for suicide bereavement. Through a 3-stage, user-centered design, merging both qualitative and quantitative data from a large sample of participants with lived or professional experiences, we were able to develop an adaptive and interactive website offered to French-speaking people bereaved by suicide. The results of the SUS during the rigor cycle and the first data regarding the use of the platform since its implementation highlight the relevance of our online resource to fulfilling the needs and expectations of French people bereaved by suicide.

### Discussion of Results

First, our results confirmed that a great proportion of people bereaved by suicide used digital resources for their grief process and reported high levels of need and expectation regarding the development of dedicated digital resources. This finding is consistent with previous studies on suicide bereavement [9] and bereavement after other causes [31], which showed high levels of use of digital resources by bereaved people.

Second, it is notable that it was decided to create a website rather than a social media platform, while 57.9% (232/401) of participants reported in the relevance cycle that they would expect dedicated social media to be developed. The choice not to create a social media dedicated to suicide bereavement was based on arguments that (1) more participants expected a website to be developed (281/401, 70.1%) than a social media platform; (2) there was a lack of existing data on the effectiveness of social media for people bereaved by suicide; and (3) there were concerns regarding the moderation of such a resource, including the risk of inappropriate behaviors between members. However, social media offers a new avenue for the grieving process [30-33], and further studies are needed to better understand how to use social media effectively and safely after suicide bereavement. Furthermore, the development of a chat dedicated to people facing suicidal behaviors, including suicide bereavement, is currently ongoing in France.

Third, we did not include any memorial pages on the website, while a substantial number of the participants in our sample reported memorialization as a critical need for digital resources after suicide bereavement [30], which is consistent with previous studies [34]. As proposed by Morley et al [35], experience-based co-design must also be based on scientific evidence from previous literature, which they term “evidence-informed experience-based co-design.” In light of the dearth of empirical evidence substantiating the efficacy of online memorials in providing assistance and support to individuals who have lost a loved one to suicide, as well as the emerging literature indicating potential adverse effects of such memorials on the well-being and mental health of those who have experienced such a loss, our decision not to develop a memorial was informed by these considerations. Westerlund [11], for example, reported that higher online support group activity predicted more satisfaction with current psychosocial health, while memorial websites seemed to have the opposite effect in a sample of Swedish people bereaved by suicide. However, other studies on bereavement by all causes have reported some positive effects of participation in online memorials [34,36,37], including the ability to foster emotional expression and continuing bonds with the deceased. Thus, implementing a memorial on the espoir-suicide website may represent an interesting avenue for which a new user-centered study is needed.

Fourth, our development study did not include any effectiveness measures after the implementation of the resources. The next step will be to develop a study aimed at measuring the effectiveness of the espoir-suicide website for fulfilling the needs of people bereaved by suicide and for improving their mental health. Further studies would benefit from examining the use of the website (including user satisfaction and use patterns) and its impact on bereavement experience, grief symptoms, depressive or anxiety symptoms, as well as on well-being and posttraumatic growth. Several questions will be raised regarding the most relevant means to assess the effectiveness of an online platform for people bereaved by suicide, which will also require the active involvement of people bereaved by suicide. According to our results, we can conclude that further studies must further explore codevelopment by identifying people with lived experiences in other domains and stages of the research, such as the design of the study, the definition of the research question, or the identification of the most relevant outcomes. Although our use of a codevelopment and user-centered design showed its relevance, the effectiveness of the website may have been even greater through the implications of people with lived experience in other stages and domains of the study.

Finally, it is notable that the espoir-suicide website was designed as a general resource for people bereaved by suicide that insufficiently considered several specific populations, including children and adolescents, which are known to display specific needs for digital resources after suicide bereavement [38]. While several pages are dedicated to the needs encountered by children and adolescents and their parents and families, after suicide bereavement, no specific space has been developed for them. A focus on specific populations that may display specific characteristics and needs [39] will have to be developed and implemented on the espoir-suicide platform in the future using a user-centered approach.

### Limitations

We performed the first user-centered study on the use of a digital resource for suicide bereavement and codeveloped the first evidence-based online resource for French people bereaved by suicide. The large sample of participants, which included a vast proportion of people with lived experience of suicide bereavement, was the main strength of our study.

However, our study has several limitations. First, the study included only French people; therefore, the generalizability of our findings must be considered cautiously. Although the results from the relevance cycle showed similar patterns to those from other countries, further studies should be conducted in low- and middle-income countries. Second, the participants were mainly women in the 3 stages of the study. Although this limitation is frequently observed in research on suicide bereavement, this issue raises the question of the ability of the espoir-suicide website to suit men bereaved by suicide. Third, due to French research legislation, we could not assess the ethnicity, religion, or sexual orientation of the participants; therefore, we cannot conclude on the ability of espoir-suicide to reach people from ethnic, religious, or sexual minority groups. This limitation is also observed for people with disabilities (those who are visually impaired, color blind, and so on). Fourth, selection bias may have occurred in our study, as participants who were bereaved by suicide, volunteers, or professionals who participated may have been particularly interested in the topic and may not have been representative of the overall target population. However, the inclusion of a diverse sample of people with lived experience, professionals, and volunteers provided a unique means of collecting a wide range of perspectives to ensure the reliability of our findings and the relevance of the platform.

### Conclusion

While people bereaved by suicide display high levels of need for digital resources, the use of a mixed methods, participatory, user-centered design allowed us to implement an evidence-based, innovative, and ergonomic website for people bereaved by suicide, showing good relevance for fulfilling the needs and expectations of French people bereaved by suicide. Our findings support the efficiency of codevelopment research through the ISR framework for developing digital resources for mental health, especially for suicide bereavement.

## Abbreviations

DSR: Design Science Research
ISR: Information System Research
SUS: System Usability Scale

## Data Availability

The data that support the findings of this study are available from the corresponding author, EL, upon reasonable request.
